# Nottingham Trent University and Makerere University School of Public Health partnership: experiences of co-learning and supporting the healthcare system in Uganda

**DOI:** 10.1186/s12992-016-0148-x

**Published:** 2016-03-28

**Authors:** David Musoke, Linda Gibson, Trasias Mukama, Yesmean Khalil, John C. Ssempebwa

**Affiliations:** Department of Disease Control and Environmental Health, School of Public Health, College of Health Sciences, Makerere University, Kampala, Uganda; School of Social Sciences, Nottingham Trent University, Nottingham, UK

**Keywords:** Health partnerships, Nottingham Trent University, Makerere University School of Public Health, Uganda, UK, Community health workers

## Abstract

Partnerships between developed and developing country institutions are increasingly becoming important in addressing contemporary global health challenges faced by health systems. Inter-university health collaboration such as the Nottingham Trent University (UK) and Makerere University School of Public Health (Uganda) partnership provide opportunities for working together in training, research and service delivery while strengthening health systems. This paper shares the experiences, achievements and opportunities of this partnership in co-learning and supporting the health system in Uganda. This includes a project being implemented to strengthen the training, supervision and motivation of community health workers in rural Uganda. Training and research are a key focus of the partnership and have involved both staff and students of both institutions including guest lectures, seminars and conference presentations. The partnership’s collaboration with stakeholders such as the Ministry of Health (Uganda) and local health authorities has ensured participation necessary in supporting implementation of sustainable interventions. The partnership uses several channels such as email, telephone, Skype, Dropbox and *WhatsApp* which have been useful in maintaining constant and effective communication. The challenges faced by the partnership include lack of funding to support student mobility, and varying academic schedules of the two institutions. The experiences and prospects of this growing partnership can inform other collaborations in similar settings.

## Background

The consensus amongst academics and practitioners alike that the most important determinants of health are the wider social, economic and environmental factors is not new. Modern conceptualizations of health start with prevention, rather than cure or care and the importance of a reinvigorated public health sector is regularly impressed upon us. Increasingly, single issue approaches to promoting health have come to be seen as limited. The main threats to human survival continue to be global in nature and include water shortage, global warming and pollution [1] as well as growing militarization [2] and conflict. All of these issues and others, including threats of global pandemics such as avian flu, severe acute respiratory syndrome (SARS) and, more recently, Ebola, increasingly demand both international and multidisciplinary responses. In addition, Kickbusch [3] argues the case for a new global social contract for a public health response that can begin to tackle the increasing divide between developed and developing countries. The debate in global health increasingly re-envisages health as a right to be accessed universally by all [4].

Health and health systems are interdependent, sharing health workers, technology and medicines. Health partnerships between institutions in the developed and developing world are becoming increasingly important in combating the 21st century global health challenges outlined above but more recently the focus has shifted towards the need to strengthen weak health systems in low and middle income countries. The challenge to build sustainable health systems requires the mobilization and strengthening of the workforce [5] and community health workers (CHWs) are now recognized as a core component of this strategy. Evidence shows that global health partnerships have many registered successes in reducing disease burden, mobilizing commitment and funding, and leading innovation [6]. Indeed, UK institutions including the government, non-governmental organizations, universities and individual professionals are making significant contributions towards strengthening healthcare systems in resource constrained countries [7] including Uganda.

The collaboration between Nottingham Trent University (NTU), UK and Makerere University School of Public Health (MakSPH), Uganda started informally with a visit of academics from the UK to Uganda in 2010 through long standing environmental health links. This was followed by several meetings facilitated in part by a Start-Up Grant provided to the partnership by Tropical Health & Education Trust (THET) which culminated in signing a Memorandum of Understanding (MoU) between the two universities. What we understood, even at that early stage, about each other’s work was that we both worked in completely different cultural contexts in relation to public and environmental health in the UK and Uganda. In addition, we also shared an understanding that working with communities is central to delivering good public health and primary health care. Public health at NTU reflects their position in a School of Social Sciences and is framed through a social model of health, using social theory and focusing on themes of health promotion, sustainability and community development, international political economy of health, the ‘contestedness’ of core concepts of health and healing, and critical philosophical debates in public health. The international public health curriculum attracts overseas postgraduate and doctoral students, including many from Africa. Robust links with health promotion practitioners, environmental health professionals, and community volunteers in Nottingham have led to participatory research focusing on the grassroots in local communities.

MakSPH is a leading public health training and research institution in East Africa, and has many years of experience of working in communities. The School, which is among the four schools of Makerere University College of Health Sciences, has four departments of Disease Control and Environmental Health; Epidemiology and Biostatistics; Community Health and Behavioural Sciences; and Health Policy, Planning and Management. MakSPH also hosts the Regional Centre for Quality of Health Care. It offers a Bachelors degree in Environmental Health Sciences and several postgraduate programmes such as Masters in Public Health, Masters in Public Health Nutrition, Masters in Health Services Research, and doctoral degrees. MakSPH also has several short courses aimed at enhancing knowledge and skills at various levels including in Water, Sanitation and Hygiene; Health Services Management; Advanced Qualitative Research Methods; Nutrition in Emergencies; and Public Health in Complex Emergencies.

Initially, the partnership had a strong desire to learn from each other about our work in communities, an approach Crisp [7] talks about when he discusses the need to refocus and rebalance development approaches so that they are not uni-directional as they have tended to be in the past. Therefore, the MoU enabled us to formalize, in our respective institutions, our relationship which was important to develop our long term collaboration. The MoU sets out the principles that guide the partnership which include aims and vision, involvement of strategic partners, commitment to sustainability, and roles of partners. Our partnership thus aims to explore opportunities for potential collaboration in the following areas: exchange of information on programme development and teaching methods; exchange of staff and students; development of joint research and collaborative projects; and any other areas to promote the academic interests of both institutions in research and/or teaching. This paper shares the experiences, achievements and opportunities of this partnership in co-learning, and supporting the health system in Uganda through a project strengthening the capacity of community health workers.

## Main text

### Working with community health workers in Uganda

The current focus of our community work is through a Health Partnership Scheme (HPS) project on health promotion in primary health care and public health. HPS is funded by the UK Department for International Development (DFID) and managed by THET. The aim of the work is to contribute to reducing the prevalence of communicable and non-communicable diseases among the poorest communities in Uganda. Sub-Saharan Africa suffers a dual burden of disease with communicable diseases such as diarrhea, cholera, dysentery and malaria contributing 69 % of deaths, and non-communicable diseases such as heart disease, diabetes and obesity causing 25 % [8]. Upstream interventions through the promotion of health, provision of health education, and effective health communication are important strategies in reducing these diseases. Indeed, globally there is increasing recognition of the need for health systems to return to the core principles of a strong primary care system: community participation, health promotion and intersectoral collaboration [9, 10]. However, across many countries, these principles remains the most under resourced component of the Alma Ata vision. Understanding and addressing the broader social determinants of health is also an important part of the partnership’s strategy and is clearly in line with Uganda’s Ministry of Health (MOH) goal of reducing the occurrence of such diseases as expressed in the Uganda Health Plan [11].

With these perspectives in mind, we identified community health workers (CHWs) as a critical cadre to focus efforts to contribute to improving Uganda’s healthcare delivery system. A CHW has been defined as any health worker carrying out duties related to delivering health care, trained in some way in the context of their work, and having no formal professional training [12]. CHWs have increasingly become part of healthcare systems in most developing countries due to limited numbers of formally trained professional health workers, and are recognized as being the cornerstone in the system in order to deliver strong primary care particularly in rural communities. The human resources for health crisis is one of the factors underlying the poor performance of health systems to deliver effective, evidence-based interventions for priority health problems. This crisis is more critical in developing countries like Uganda which have registered a high rate of health workers migrating to work in developed countries in recent years [10]. CHWs can add significantly to the efforts of improving the health of the population, particularly in settings with the highest shortage of motivated and capable health professionals [13, 14]. In addition, where CHWs programmes exist, they are expected to increase equity and coverage of health services compared to alternative service delivery models [15]. Studies in developing countries, including Uganda, have also illustrated cost reduction and cost-effectiveness of community based tuberculosis care offered by CHWs [16, 17].

Uganda’s health worker to patient ratio of 1 per 1298 people is one of the highest in Africa and is below the World Health Organization (WHO) minimum standard of at least 1 health worker per 439 people [18]. To bridge this gap, MOH in 2001 through the National Health Policy of 1999, the Health Sector Strategic Plan (HSSP) I 2000/01 – 2004/05 and HSSP II 2005/06 – 2009/10 introduced a CHWs programme known as the Village Health Team (VHT) strategy as part of the Uganda National Minimum Health Care Package (UNMHCP). The UNMHCP is intended to provide every village in Uganda with the capacity to mobilize individuals and households for better health [19, 20]. The VHT comprises of community volunteers who are selected from within their communities to provide accurate health information, primary healthcare support and appropriate linkages to health services. They are the first point of contact for healthcare delivery in the community and have been incorporated by MOH into the health system as Health Centre I level. However, VHTs in Uganda are faced with various challenges that include inadequate training, minimal supervision and low motivation [21]. In addition, like in many other developing countries where similar CHWs programmes exist, they are marred with high attrition levels [22] and poor performance [13]. Our partnership therefore anticipated to create a significant impact on the country’s health system by focusing on this cadre. VHTs mobilize community members and help to increase community participation in local health programmes. Other specific roles of VHTs include referring patients to health facilities, collecting household data, treating childhood diseases, conducting health education and acting as role models for community members.

### Needs assessment among community health workers in Uganda

We conducted a needs assessment in 2012, supported by the THET Start-Up Grant from the Health Partnership Scheme, to determine the situation and provide up to date information about the status of the CHWs programme in Uganda. The needs assessment involved holding nine focus group discussions (FGDs) with CHWs, three each from urban, peri-urban and rural areas. In addition, ten key informant interviews (KIIs) were held in those communities. The key informants were health assistants (three), CHWs supervisors (three) and local leaders (four). The FGDs and KIIs were conducted by an experienced researcher with support of a research assistant. This needs assessment supplemented earlier findings [21] and confirmed the existence of several challenges affecting CHWs in the country. It was established that although CHWs did a tremendous job in primary health care and public health, they experienced several challenges that affected their work. These included insufficient initiation training, limited continuous training, lack of basic necessities such as gloves, minimal incentives for motivation, limited reference materials, minimal support supervision, and the available guides being in English (yet the majority could only read the local language). The needs assessment also established that although all four CHWs per village, who were mainly selected by the community, are involved in general duties such as health education and home visiting, only two of them were involved in integrated community case management (iCCM) of childhood illnesses (malaria, diarrhea and pneumonia). For the UK partners, these results enabled them to see the challenges facing CHWs at local level. At the same time, it was also appreciated that community development programmes using CHWs often suffer from issues such as lack of funding.

### Strengthening the community health workers programme in Uganda

Based on the results of the needs assessment, we developed a proposal to strengthen the CHWs programme in Wakiso district, Uganda which received funding support from the Health Partnership Scheme (September 2014). The two and a half year project focuses on three components of the CHWs programme: training, supervision and motivation. Before commencement of implementation, the project obtained ethical approval from Makerere University School of Public Health Higher Degrees, Research and Ethics Committee which is a national requirement. The project was also registered at the Uganda National Council of Science and Technology. This contributed to ensuring that ethical aspects including the rights and interests of CHWs and other individuals participating in the project are protected.

NTU, the lead UK project partner and grant holder, works closely with MakSPH which is the lead Uganda partner responsible for the day-to-day running of activities. To enable smooth implementation of project activities, NTU and MakSPH signed a sub-contract in addition to the funder’s contract with NTU. The sub-contract stipulates the duties, expected outputs and timelines for both partners. The project is being implemented in partnership with MOH and C3: Collaborating for Health, UK. MOH being the national policy formulation institution provides support in the overall management of the project including planning and implementation of activities. C3 is involved in project management and brought to the partnership a wealth of experience of working with communities in Uganda. Wakiso district health department is involved in project planning and implementation of activities notably training of CHWs and their supervision. All partners are involved in all stages of the project which include preparation, implementation and evaluation. The preparatory phase (November 2014 to April 2015) was completed; implementation phase (May 2015 to October 2016) is currently ongoing; and the evaluation phase (November 2016 to April 2017) will be conducted on completion of implementation of activities.

The preparatory phase included a baseline survey which assessed the functionality of the VHT programme including knowledge, skills and practices of CHWs. This survey was conducted among all the CHWs in Ssisa sub-county, Wakiso district where the project is being implemented. Review of existing literature on CHWs in Uganda as well as related MOH policy documents were also reviewed. The survey established that out of the 300 CHWs to be targeted by the project, only 191 (63.7 %) were functional hence active. The non-functionality of many CHWs was established to be because of not having had an opportunity to be trained so as to be able to carry out their duties, and others who had initially been trained having dropped out of the programme. From interaction with the supervisors of CHWs, there was a higher drop-out rate of CHWs not involved in iCCM because they had received less attention from national programmes including in terms of training, supervision and motivation. The majority (95.8 %, 183/191) of active CHWs reported receiving initiation training on their work which was conducted in 2010. However, a third (32.8 %, 60/183) of them had not undergone any other training besides the initiation training. The supervisors of CHWs reported lack of transport as the main challenge in performing their duties which included delivering supplies to CHWs, and collecting monthly reports and delivering them to health facilities. Despite their key role in the CHWs programme, supervisors noted that they had received minimal training regarding their supervisory duties. This was evident given the fact that many of them could not appropriately complete the supervisory forms that were provided to them.

The majority of CHWs reported having received some non-financial incentives including t-shirts 97.4 % (186/191), bicycles 24.6 % (47/191), bags 87.4 % (167/191) and badges 83.3 % (159/191). However, few CHWs still had these items at the time of the survey and majority were worn-out since most of them had been received in 2010 without replacement. Although all the CHWs had never received gumboots and umbrellas, it was established that these items were necessary in performing their work especially in the rainy season. Indeed, the CHWs stressed the importance of the various incentives in carrying out their duties. Whereas most (98.4 %, 188/191) of the CHWs owned mobile phones, almost half (49.5 %, 93/188) charged them from commercial places, often characterized by travelling long distances to and from the charging points. The findings of the baseline survey further emphasized the gaps in the CHWs programme in Ssisa sub-county, Wakiso district hence the need for support. The partnership’s project, with a focus on training, supervision and motivation of the CHWs, was designed to support the work of the CHWs hence enhance their performance for health improvement in the community.

The intervention phase of the project is focused on developing training materials, holding training sessions for both CHWs and their supervisors, enhancing transportation of supervision through provision of motorcycles, and providing non-financial incentives of umbrellas, solar chargers, gumboots and t-shirts to motivate the CHWs. The training of CHWs is adapted to the needs of the trainees, job and tasks they are expected to perform, and the context in which they are working. Supervision of CHWs, supported through training of supervisors and provision of motorcycles, has provided an opportunity for CHWs to regularly share individualized performance needs as well as discuss issues pertaining to their work with the supervisors.

The project has trained 24 supervisors of CHWs for 1 day on their roles and responsibilities, communication skills, management, data collection, reporting and record keeping. In addition, 301 CHWs have been trained for 2 days on their roles, communication, record keeping, home visiting, health promotion, first aid, community mobilization, child growth and common diseases. Among these CHWs, 136 involved in iCCM underwent an additional 2 days of training on use rapid diagnostic tests (RDTs) for malaria diagnosis, reporting using mobile phones (mTrac), and management of childhood illnesses. The trainings have all been conducted by local health practitioners who have been oriented by the project. All trained CHWs have received t-shirts, umbrellas and gumboots as part of the training and motivation package provided by the project. Among the CHWs, 75 who were at most need received solar chargers to facilitate charging their phones so as to enhance communication. CHWs in villages that do not have electricity and those involved in iCCM who use their phones for reporting were given priority while selecting those to receive the chargers. Three motorcycles have also been provided by the project to support transportation of supervisors of CHWs during their supervisory work. The motorcycles have been greatly appreciated by the community and are already enhancing supervisors of CHWs in delivering drugs and supplies to them as well as collecting reports during supervisory visits.

Project evaluation involves routine monitoring of the performance of CHWs at regular intervals and will include a mid and end of term evaluation. The main performance indicators of the project are number of people health educated and households visited (for all CHWs), and number of children under 5 years of age treated (for only CHWs involved in iCCM). This data is collected by the trained CHWs using their reporting forms which is then collected from them every month by their supervisors. So far, the CHWs trained by the project have made 11,149 household visits, health educated 32,295 people (17,994 females and 14,301 males), and treated 1884 children against malaria, diarrhea and pneumonia. As more CHWs are trained, more households and individuals are being reached during the various activities of these health volunteers.

### Research and training

The partnership has enhanced research and training in the two institutions. Two Master’s students from NTU have conducted their graduate research projects in Uganda. Their research projects focused on examining how CHWs in Uganda understand ‘participation’ as a concept in practice [23]; and what the perceived barriers to early breast cancer detection in Wakiso district are amongst CHWs [24]. The students received support in the field from staff and students at MakSPH. They also volunteered in on-going partnership activities in Uganda such as workshops, seminars and conferences. In addition, members of NTU have made several presentations over the period of the partnership at the Makerere University Environmental Health Students’ Association (MUEHSA) conference organized annually by students of Environmental Health at MakSPH. This has increased the opportunities for transferring knowledge and sharing research findings between the two countries. The participation of UK partners in MUEHSA conferences plays a major role in enhancing inter-disciplinary knowledge about global public health issues for the Ugandan students. NTU and MakSPH staff have also made several guest seminar presentations during their respective visits to host institutions. Staff members of MakSPH have also participated in guest class lectures at NTU on Masters programmes, two of which were conducted via Skype to discuss issues of community participation from the field experience of the researchers. Our partnership base has been broadened by working with a Nottingham based health promotion practitioner with extensive experience in community development, monitoring and evaluation. Support for the partnership from the MOH, CHWs representatives and local health authorities is critical in the implementation of various research activities, and the building of trust and reciprocity underpins our activities in the field.

### Strategies

We have always conceptualized the relationship between NTU, MakSPH and other partners as one of mutual respect and learning from each other. From our experience, trust among partners, with volunteers and community members takes time. MakSPH’s long term working relationship with local communities including where partnership activities are based has been profound in fostering trust with local communities and responsible authorities.

Community development as an approach to working with excluded communities is a key component of our activities. We listen to the needs of the people and develop with them solutions that work and are sustainable in the long term beyond the life of projects. By using various techniques of communication and participation for designing the different components of interventions, and for building capacity within the community, our work aims to be owned by the communities we work with in the long term. Part of this is building partnerships with various stakeholders for example MOH, health facilities and local health authorities that can be sustained.

### Communication

NTU and MakSPH have been communicating during the duration of the partnership both formally and informally. We have developed an understanding of each other’s work and roles which has allowed us to develop our ideas and thinking incrementally. This has also enabled us easily build up trust between each other as well as an understanding of how our institutions work including their relationship with local communities. Although the context in each country is very different, we have been able to develop shared dialogue, communication, understanding and ethos. Communication globally is now easier due to mobile technology and social media. Indeed, most of our communication is through email, Skype, telephone and *WhatsApp. WhatsApp* has particularly been useful for short messaging in cases where we have had to respond to issues quickly. Telephone calls are used in situations when internet connection is poor, especially in Uganda. In addition, Dropbox has been very useful for sharing big files such as photographs and reports. The UK staff and volunteers also write blogs about their work during visits to Uganda and this has been very useful and accessible for disseminating tales from the field. In addition, articles are written about major partnership events and published in institutional newsletters or posted on university websites to raise awareness and profile with our institutions and beyond.

### Challenges

For the UK team, drawing from the student body to develop opportunities for volunteering presents some challenges for the future. Funding is a major challenge, but we are set to explore the Erasmus Plus programme which in 2016 will extend to include Africa in its opportunities for student exchange and other activities. The other challenge is building cultural competence and resilience to work and live in a culture that is very different. Our first student volunteer was able to accompany NTU staff and mentoring was easy. Local students in Uganda are very enthusiastic in supporting UK volunteers and we are thinking about building a buddy system so they can be paired up in advance. Another challenge faced by the partnership is the different academic schedules which at times affect planning and implementation of activities. In addition, the academic programmes of each institution have been developed using different curricula hence the difficulty in introducing collaborative components in existing programmes. Nevertheless, the partnership is looking at developing new programmes that take into account the interests of both institutions.

### Future opportunities

The partnership is exploring several opportunities to expand activities and involve other partners in its work. Meetings have been held with Change Makers, who are community health volunteers in Nottingham, so as to explore opportunities to collaborate with them. Plans are underway to link the Change Makers with Ugandan CHWs which will support sharing of experiences and learning from one another. NTU and MakSPH are planning co-edited books which will contribute to the body of knowledge on global public health. An international symposium on patient safety is also being planned by the partnership, and more resources are also being sought to enhance staff and student mobility between the UK and Uganda in future. Collaborative research projects are being developed between NTU and MakSPH to further the partnership’s research agenda. Discussions are also underway to explore collaborative training programmes including summer schools, and joint supervision of PhD students. As a partnership, we have taken a step by step approach to our collaboration as we increasingly develop our understanding of each other and expand our networks of collaboration with the community, policy makers, academics and students. We therefore see our activities expand in what we envisage to be a sustainable and integrated manner to the mutual benefit of all.

## Conclusion

This collaboration between NTU and MakSPH was borne out of a shared understanding of the importance of communities in delivering strong health promotion and primary health care which strongly informed the CHW project that we developed. The partnership was also made up of a commitment to the principle of valuing different knowledge and expertise. Each partner had different strengths: in the UK the focus is on the social model and broader determinants of health whilst in Uganda core strengths were environmental, public and community health. In addition to the commitment and expertise of staff and students involved, exchange visits, workshops and seminars were also supported by a strong commitment of our respective institutions in both countries to sustain the partnership—even when there was no available funding. This commitment to the value of shared learning means that students, staff and volunteers benefit from this inter-disciplinary approach to knowledge exchange.

The partnership also demonstrates how leveraging international expertise and local knowledge can support the health system in a developing country including CHW training, research and provision of services. Our collaboration is exploring how to scale up interventions in the competitive funding environment, remaining open and adaptive to supporting communities in resource poor settings, and to continue working with key actors on implementation of policy from an upstream, preventative perspective to achieve the right to health for all. The achievements, experiences and prospects of this growing partnership can inform other collaborations in similar settings.

## Abbreviations

CHWs: Community Health Workers
DFID: UK Department for International Development
FGDs: focus group discussions
HPS: Health Partnership Scheme
HSSP: Health Sector Strategic Plan
iCCM: integrated community case management of childhood illnesses
KIIs: Key informant interviews
MakSPH: Makerere University School of Public Health
MUEHSA: Makerere University Environmental Health Students’ Association
MOH: Ministry of Health
MoU: Memorandum of Understanding
NTU: Nottingham Trent University
THET: Tropical Health & Education Trust
UNMHCP: Uganda National Minimum Health Care Package
VHT: Village Health Team
